# Two methods, two views: Integrating phytoliths in thin sections and bulk samples on the urban Dark Earths from the DIVA-site (Antwerp, Belgium)

**DOI:** 10.1371/journal.pone.0320122

**Published:** 2025-03-31

**Authors:** Mónica Alonso-Eguiluz, Sarah Lo Russo, Luc Vrydaghs, Pascal Tribel, Gianluca Bontempi, Arnaud Schenkel, Daan Celis, Karin Nys, Yannick Devos

**Affiliations:** 1 AMGC- Archaeology, Environmental changes and Geo-Chemistry, Vrije Universiteit Brussel, Brussels, Belgium; 2 IPAS - Integrative Prehistoric Archaeological Science at University of Basel, Basel, Switzerland; 3 Machine Learning Group (MLG), Université Libre de Bruxelles, Brussels, Belgium; 4 PANORAMA, Université Libre de Bruxelles, Brussels, Belgium; 5 Urban Archaeology Department, City of Antwerp, Antwerp, Belgium; Universita degli Studi di Milano, ITALY

## Abstract

Traditionally, phytolith analyses are carried out by extraction from bulk (sediment) samples. This technique provides valuable information, not only on the morphological and/or taxonomic assignment of phytoliths, but also on their concentration (quantitative analysis). However, extraction leads to the loss of the (micro-)context in which they are embedded. Over the past 20 years, the study of phytoliths in soil thin sections has proven to be a consistent method. As phytoliths are neither removed from their sedimentary matrix nor artificially concentrated, their analysis provides information on their taphonomical history, but their morphological identification is sometimes limited. Therefore, it seems obvious that the next step to improve phytolith analysis is to combine the two approaches. The aim of this paper is to explore the potential of this integration. For this purpose, we focus on the urban Dark Earth of the DIVA-site (Antwerp, Belgium), with a chronology between the end of the Gallo-Roman Empire and the 11th century AD. Three different stratigraphic units, micromorphologically recognized within the Dark Earth, have been studied. They correspond to an agricultural field, unconsolidated walking surfaces and a floor. Our results confirm the added value of combining the two methods. The possibility of observing the phytoliths in their (micro-)context allowed us to characterize each stratigraphic unit with a particular phytolith assemblage. At the same time, the information derived from the bulk samples overcomes the difficulties in the morphological identification of phytoliths in soil thin sections.

## Introduction

Silica phytoliths (hereafter phytoliths) are biomineralizations produced within the tissues of the living plants. Along with water, plants absorb monosilicic acid (Si(OH_4_)), which is transported to different organs and tissues and silicifies in the cellular structure of the plant tissue, reproducing the cell shape [1]. Phytoliths are deposited in soils or sediments, either within part of the plant material or released from organic matter, leaving a botanical signal that provides local ecological information [2,3]. Traditional methodological approaches for phytolith analysis are based on the collection of bulk samples (BS) and their subsequent laboratory treatment for phytolith extraction. This method provides important quantitative information that can be used to assess natural or anthropic plant input and preservation issues in paleoenvironmental and archaeological contexts [4–9]. However, phytolith analysis in BS necessarily implies the loss of important (micro-)contextual information [10,11]. The collection of sediment samples, and the extraction procedures in the laboratory, normally carried out by ashing and/or acid treatment, are the two main processes involved in the loss of that (micro-)context. Hence, to observe phytoliths within their microscopic context, phytolith studies must be conducted on undisturbed samples, e.g., soil and sediment thin sections (STS). The study of the distribution patterns of phytoliths within STS permits us to understand the depositional and/or postdepositional history of the phytolith record [10,11]. Nevertheless, there are some limitations to the study of phytoliths in STS, such as the difficulty of identifying certain morphotypes, as phytoliths are cut at random angles and cannot be rotated; the potential poor visibility caused by the fine fraction, which can partially or completely mask microremains [10,11]. Although it is not a generalized method, the study of phytoliths in STS has been applied systematically to the study of the urban Dark Earths in Belgium [3,12–17], providing valuable information on the ancient use of plants.

Bearing in mind that both techniques have advantages and limitations, it becomes crucial to combine these techniques to fill the gaps of both methods. The aim of this paper is to discuss how the integration of phytolith analyses in BS and STS complement each other and to evaluate the potential of the resulting data. To achieve those goals, a pioneering study integrating phytolith analyses in BS and STS has been realized on the archaeological site of DIVA, situated in the historical center of Antwerp (Belgium), where several meters of urban Dark Earths were exposed during excavations [18].

The medieval origin and development of Antwerp represents a challenge for archaeologists and historians, especially the period bracketed between the end of the Roman empire and the 11^th^ century, due to the scarcity of written documents [17,19,20]. Hence, archaeological evidence becomes a critical source of information regarding the activities carried out in the city of Antwerp during these centuries. In this respect, the DIVA-site is of particular interest as it covers this poorly documented period. During excavation, the archaeologists discovered meters thick urban Dark Earth. These Dark Earths are thick dark colored homogeneous archaeological deposits, ubiquitous in urban areas throughout Europe [21]. Their micromorphological study permitted to document agricultural and domestic activities (walking surfaces and floors) on the DIVA-site [18] thus offering the opportunity to study different types of human activities. Micromorphological descriptions of the samples analyzed are given in Table 1.

**Table 1:** Table summarizing the micromorphological interpretations. Raw materials of the type of deposit are available on I-GEOARCHive.

Samples	Main characteristics	Type of deposit
196.3, 196.4, 196.5	- Microscopic traces of soil working, dusty clay coatings, strongly fragmented and randomly distributed anthropogenic remains - Soil enrichment, including ceramics, earthen construction material, calcareous ash, charcoal and bone - Anomalous high bioturbation	Agricultural field (AF)
196.3	- Predominant horizontal orientation of components: building material, ceramic fragments, fragments of volcanic rock (possible millstones), charred and heated material, organic material) - High bioturbation - *In situ* fragmentation of components	Walking surfaces (WS)
196.1	- Channel and platy microstructure - Very organic layer with locally a clear horizontal orientation of the organic components	Floors (FL)

## Materials and methods

### Thin sections

**Fig 1:**
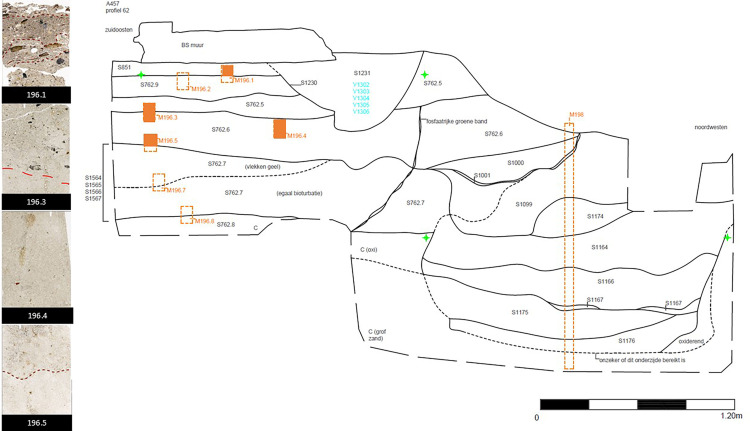
Profile 62 of the archaeological site of DIVA (Antwerp). The studied thin sections are shown on the left, and their provenience in the profile is marked in orange.

Three types of deposits from profile 62 defined by micromorphology [18] (Table 1) were selected for this study (Fig 1):

- An agricultural field (AF) observed in samples 196.3, 196.4 and 196.5;
- A walking surface (WS) observed in sample 196.3;
- Floor levels (FL) observed in sample 196.1.

The field site access for sampling was granted by the Urban Archaeology Department, City of Antwerp, Belgium.

The late Ing. T. Beckmann (Germany) manufactured the 30 µm-thick thin sections (6x8 cm). STS were analyzed at the Vrije Universiteit Brussel under a ZEISS Aixoscope 5 petrographic microscope equipped with fluorescence light. After the micromorphological study was completed, phytolith analysis took place based on the micromorphological descriptions. Phytolith observations were performed on between 11 and 22 squares of 5x5mm selected from each studied unit. Within each square, four fields of 0.2 mm^2^ were systematically analyzed under PPL, XPL, UV and blue light at 500x [14]. In order to test whether the phytoliths were fluorescent and had therefore been burnt, they were examined under UV and blue light [22,23]. To describe phytoliths in STS the methodological approach developed by Vrydaghs and Devos was followed [10,14]. This method considers the distribution patterns of phytoliths and their VPC index: visibility, preservation, and color. The distribution pattern refers to how phytoliths may appear in soils and sediments: isolated, clustered (several phytoliths with no anatomical connection and different orientations); or articulated (interconnected phytoliths maintaining the original anatomical position they had in the plant tissue). Regarding the VPC index, visibility is described as perfect (A), good (B), moderate (C) or bad (D). Preservation is described as perfect (A), almost perfect (B), good (C), moderate (D) and bad (E). Finally, phytolith color is described according to two categories: colorless (A) and colored (B) [10].

Additionally, morphometric analyses were performed on two articulated systems from FL following Vrydaghs et al. and Ball et al. [3,24]. Morphometric analysis was carried out by measuring the size and shape of more than 30 wave lobes [24]. Results were compared to the measurements data set from a reference collection that includes the data of 49 taxa distributed over 156 specimens collected following Ball et al. [25].

### Bulk samples

Three sediment BS were collected from profile 62, from each context, FL, WS and AF. Phytolith extractions were carried out following the methods of Katz et al. [26]. Between 20 and 50 mg of sediment was placed in a 0.5 ml Eppendorf tube and 50 µl of HCl 6N was added. After the reaction, 450 µl of sodium polytungstate solution (SPT) [Na6(H2W12O40) ∙ H2O] with a density of 2.4 g/ml was added. The tube was vortexed and sonicated for 10 minutes and then centrifuged for 5 minutes at 5000 rpm. The supernatant liquid was removed and transferred to another tube. 50 µl of the aliquot was placed on a microscope slide and covered with a 24x24 mm coverslip. BS were analyzed at the Royal Belgian Institute of Natural Sciences, under a ZEISS microscope. Phytoliths present in 20 visual fields at 200x were counted for phytolith quantification, and morphological identification was carried out at 500x.

### Phytolith inventory

Morphological identification of phytoliths in BS and STS was based on modern reference collection (www.phytcore.org) as well as standard literature [1,27]. The nomenclature of the phytoliths followed the International Code for Phytolith Nomenclature 2.0 [28], whenever possible. A minimum number of 200 recognizable phytoliths were identified to obtain a reliable phytolith morphological interpretation [4]. Whenever possible, morphotypes were grouped into different categories based on their taxonomic and anatomical provenance: Poaceae (C_3_ and C_4_), Poaceae leaves/stems, Poaceae inflorescence, dicots wood/bark, dicot leaves. Elongate entire is the only morphotype which was not included in any of these groups, as it is commonly produced in the leaves and stems of grasses and sedges, and it can also be found in dicotyledonous plants in lesser numbers [28].

Phytoliths showing bad preservation were classified according to three categories: fragments, resulting of mechanical processes; weathered phytoliths, suffering severe chemical alteration and the subsequent loss of their morphological attributions, making them unidentifiable; and melted phytoliths, presenting evidence of burning such as change in color (darker color) and bubbles on their surface. If thermal alterations are too extreme phytoliths and other silica microremains lose their autofluorescence and morphology appearing as vitrified silica [23,29–31].

## Results

### Phytoliths in soil thin sections

This section focuses on the phytolith descriptions of the STS. First, the distribution patterns and the VPC ratio are described. Subsequently, the morphotypes inventory is detailed.

#### Distribution patterns.

**Fig 2:**
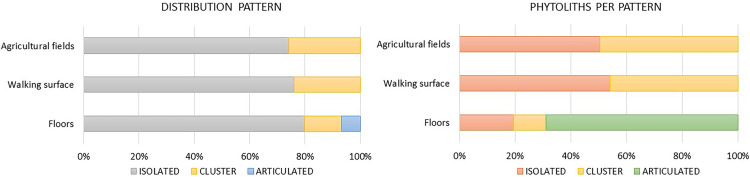
Plots showing the percentage of distribution patterns per sample and the percentage of phytoliths present in each pattern.

Table 2, S1 Table and Fig 2 illustrate the distribution patterns documented in the STS. Overall, isolated and clustered phytoliths are the most abundant patterns and are observed in all the studied stratigraphic units. Between 74% and 80% of the assemblage consists of isolated phytoliths. (Table 2, Fig 2). Clusters represent between 14% and 26% (Table 2, Fig 2). Finally, articulated systems were observed only in the FL, albeit in low amounts (6%) (Table 2, Fig 2). The articulated systems show horizontal to (sub)horizontal orientation and are mostly surrounded by organic remains.

**Table 2:** Absolute values of the number of distribution patterns and of the total amount of phytoliths identified in the patterns, described in each of the stratigraphic units.

	AGRICULTURAL FIELDS		WALKING SURFACE		FLOORS	
	Distribution pattern	Phytoliths per pattern	Distribution pattern	Phytoliths per pattern	Distribution pattern	Phytoliths per pattern
Isolated	446	446	99	99	75	75
Cluster	149	417	31	84	13	48
Articulated	–	–	–	–	6	266

Regarding the amounts of phytoliths per distribution pattern, in AF and the WS, between 50 and 54% of the phytoliths appear isolated, while phytoliths in clusters constitute between 46 and 49% (Table 2, S1 Table, Fig 2). FL are the exception to this trend, since the 68% of the phytoliths described appear in articulated systems, and the percentages of isolated and clustered phytoliths is less than 19% (Table 2, S1 Table, Fig 2).

#### VPC index.

**Fig 3:**
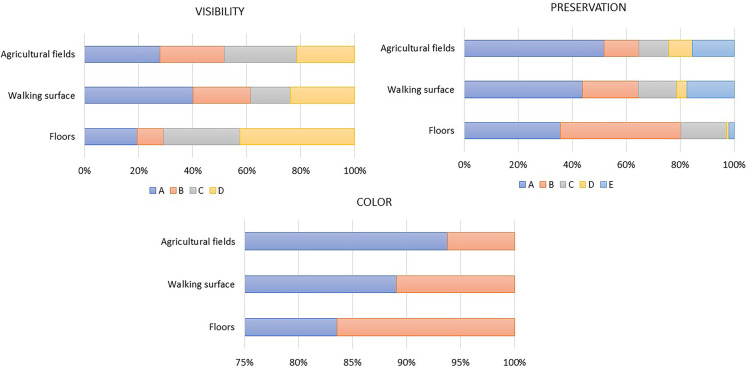
Plots showing the percentages of each of the categories of the VPC index for each of the stratigraphic units analyzed in this work.

*Visibility*. Visibility in AF and WS is quite similar with almost the 60% of perfect to good visibility (A and B). Moderate visibility (C) is bracketed between 14 and 24% and bad visibility (D) shows similar amounts established between 18 and 23% (S2 Table, Fig 3).

Conversely, these values change completely in FL. 19% of the phytoliths present a perfect visibility (A). Good visibility (B) has the lowest presence, 10%. Moderate visibility (C) represents 28% of the phytoliths. Finally, bad visibility (D) rises to 42% (S2 Table, Fig 3).

*Preservation.* There is an overall good phytolith preservation in all the stratigraphic units, although we can observe some differences between them. Perfect preservation (A) was documented in percentages between 35 and 50%. Almost good preservation (B) shows a relative amount between 14 and 20% in AF and WS, while its presence is more abundant in FL, 44%. Good preservation (C) has similar amounts in the three stratigraphic units, bracketed between 12 and 16%. Values of moderate preservation (D) are the lowest in the three cases, ranging from 1 to 7%. Finally, the most remarkable difference is found in the bad preserved phytoliths (E), which are more abundant in AF and WS, between 15 and 17%, while the presence of bad preserved phytoliths is scarce in FL, 2% (S2 Table, Fig 3). Weathered phytoliths were scarce to absence in all the samples: < 1% in FL; 4% in WS; 6% in AF (S2 Table, Table 3). Conversely, fragmentation is widely documented in AF and WS, 7 and 10% respectively, while in FL fragments only represent 1.2% (S2 Table, Table 3).

**Table 3:** List of phytoliths identified in STS and the percentage of weathered morphotypes, fragments and melted phytoliths. Other silica microremains as the percentage of diatoms and sponge spicules are also listed.

	Phyts per mm^3^	#Phyts identified	%Weathered phytoliths	%Fragments	%Auto fluorescent phytoliths	%Melted phytoliths	%Diatoms	%Sponge spicules
Agricultural fields	70,000	586	6	7	1%	<1	1	1
Walking surface	44,000	183	4	10	3%	<1	3	2
Floors	47,000	389	<1	1	3%	–	1	–

In terms of concentration per mm^3^, it can be observed that AF represent an almost double concentrations compared to FL and WS, with 70,000 vs 47,000 and 44,000 phytoliths per mm^3^ respectively (S2 Table, Table 3). More importantly, however, is the huge variety that is attested between the different studied fields of observation (Table 4). FL shows the highest variability since phytoliths were not observed in some of the fields, while others are extremely rich, showing several hundreds of phytoliths (Table 4).

**Table 4:** List of the stratigraphic units analyzed alongside with the number of fields observed, the maxim and minimum number of phytoliths observed in one field, the mean and the standard deviation.

Unit	#Fields	Maximum # of phytoliths observed in one field	Minimum # of phytoliths observed in one field	Mean	Standard deviation (s)
Agricultural fields	48	11	1	6	3.5
Walking surfaces	10	11	1	6	3.5
Floors	20	158	–	79	55.8

*Color.* The vast majority of the phytoliths were colorless (85%), the highest percentage of colored phytoliths (B) is found in AF (7%) (S2 Table, Fig 3). Although not always a defining condition, colored phytoliths are usually associated with combustion processes that change the color of these microremains [23,30–33].

**Fig 4:**
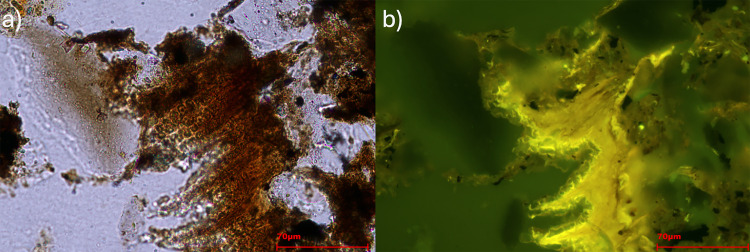
Microphotographs taken at 500x under PPL (a) and blue light (b) of an articulated system formed by Elongate dentate from FL. Note that organic matter is partially masking the articulated system, making the observations difficult. Under blue light, organic matter is auto-fluorescent.

**Fig 5:**
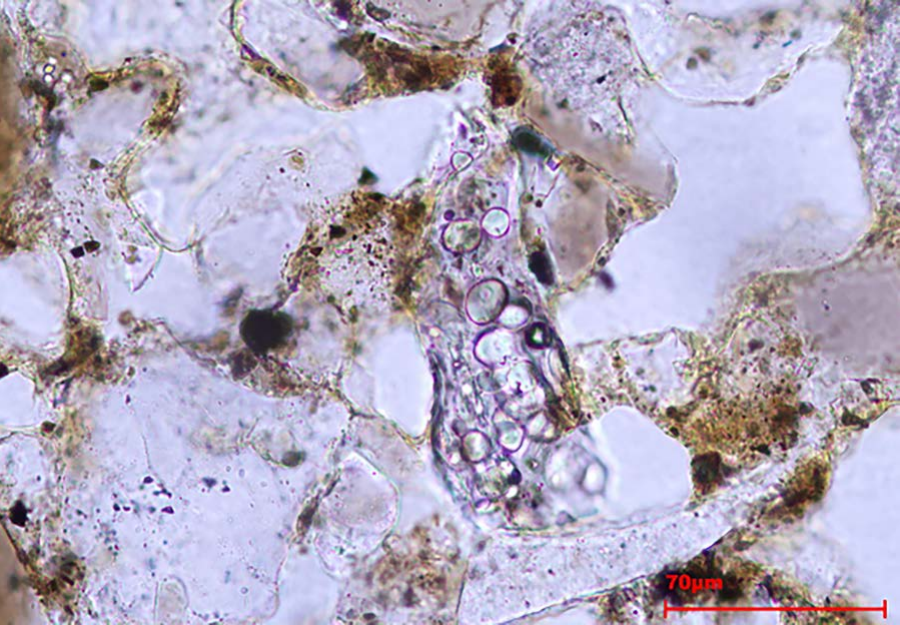
Microphotograph taken at 500 magnifications of an isolated melted phytolith (AEB) under PPL.

Fluorescent phytoliths are present in WS (3%) and FL (3%), while they are scarce in AF (1%). Additionally, in the case of the FL, auto-fluorescent phytoliths were not observed within the articulated systems (Fig 4). Thermal alterations (change on the color of the phytolith, bubbles on the surface) were also documented, although in minor amounts (<1%) in the AF and WS (Fig 5, Table 3).

#### Phytolith morphological analysis.

**Fig 6:**
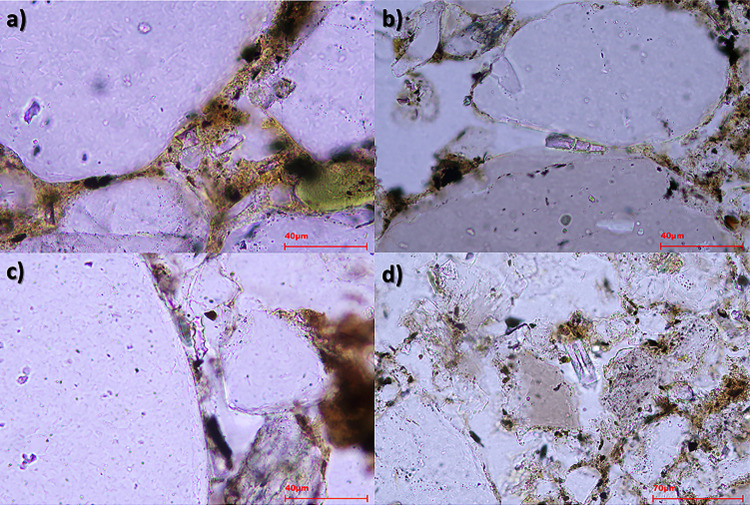
Microphotographs taken at different magnifications under PPL: (a) Isolated GSSCP Rondel (AAA) taken at 80x from STS 196.5; (b) Isolated Acute bulbosus (ACA) at 80x from STS 196.5, note that the phytolith is fragmented into three pieces and slightly moved as there is space between the pieces; (c) Isolated Elongate dentate (ACA) taken at 80x from STS 196.4; d) Isolated spicule (BCA) taken at 50x from STS 196.5.

Table 5 lists the 19 morphotypes identified alongside their taxonomical and anatomical attribution. Monocot plants are the most abundant in the three stratigraphic units, particularly Poeaceae (grasses) which have been documented through the presence of the so-called *grass silica short cell phytoliths* (GSSCP) and elongate with different margins features. GSSCP morphotypes are produced in the epidermal tissue of grasses and have a high ecological value [25,32]. The most abundant GSSCP morphotypes identified in the DIVA samples are those produced by C_3_ Pooideae subfamily plants, especially Rondels (Fig 6a) (<34%), along with Trapezoid (<4%) and Crenate (<1%) (Table 5). Plants with a C_3_ photosynthetic pathway grow in high latitudes and temperate to humid environments [27]. Also, the most important crops such as wheats, barleys, rye, or oat belong to this subfamily [34]. Besides these morphotypes, Bilobate was also documented in AF and WS, although to a lesser extent (<1%) (Table 5). This morphotype is widely produced by Panicoideae grasses, usually found in tropical and subtropical areas [27,35] to which millets (i.e., *Panicum milliaceum, Setaria italica*) belong to. Whilst Bilobate can be produced by C_3_ plants as well, as is the case of the Arundinoideae subfamily (reeds) [36,37] or the plants belonging to the Stipeae tribe (Pooideae) (i.e., *Stipa avenaceae*) [38], which grow in open environments.

**Table 5:** Percentage of phytolith morphotypes identified in the STS of the three stratigraphic units, along with their botanical attribution.

Morphotype	Plant attribution	Agricultural fields	Walking surfaces	Floors
Elongate entire	Elongate entire	22	18	5
Elongate with margin features	Grasses	1	–	1
Rondel	Pooideae	26	34	11
Trapezoid		4	2	<1
Bilobate	Pooideae/Panicoideae	<1	1	–
Acute bulbosus	Grass leaves	1	3	1
Bulliform		–	<1	<1
Crenate		2	<1	<1
Elongate sinuate		2	3	–
Elongate dendritic	Grass inflorescences	2	4	51
E. dendritic/dentate		2	4.4	21
Elongate dentate		<1	1	1
Papillate		<1	1	–
Papillate base		–	–	1
Cone shape	Cyperaceae	<1	–	–
Blocky	Dicots wood/bark	<1	<1	–
Spheroid		<1	–	–
Elongate thick	Dicots leaves	–	1	–
Indeterminable		34	25	6

Morphotypes produced by grass leaves observed are Acute bulbosus (<3%) (Fig 6b), Elongate sinuate (<3%), and Bulliform, although to a lesser extent (<1%) (Table 5). Grass inflorescences have been documented through the presence of Elongate dendritic, E. dentate (Fig 6c), along with Papillate and Papillate base. The presence of E. dendritic is important as it is indicative of crops, especially if its presence rises above 8% [39]. The greatest amount of E. dendritic was found in FL, where its occurrence rises to 51% (Table 5) and has been mainly observed in articulated systems. Unexpectedly, in samples from AF this morphotype is scarce, < 2% (Table 5) and they appear isolated and in clusters. In addition, in many cases it is not possible to distinguish between E. dendritic and dentate, due to their orientation, visibility or a combination of both. In those cases, we refer to them as Elongate dentate/dendritic, but we do not consider them as indicative of crops. E. dentate/dendritic where identified in all the samples, but in the case of FL they appear in a 21% (Fig 6c, Table 5).

Morphometric analyses carried out in one of the articulated systems were not conclusive, although there is a trend for *Avena sativa*. Also, descriptions conducted in light microscopy on isolated dendriforms deriving from botanical specimen: *Avena sativa L.* (syn. A. Byzantina), common oat; *Bromus intermedius* Guss.; *Bromus tectorum* L., cheat grass; *Hordeum* sp., barley; *Triticum durum* Desf. (syn. T. pyramidale), durum wheat; suggest that morphological criteria allow to differentiate between species. Our observations noticed that E. dendritics from these articulated systems present an angular triangular apex, and in the side view circles are inscribed in the dendriform outline, which is comparable with the one presented by Avena sp. [12].

Along with grasses, other monocots have been observed. This is the case of Cyperaceae (sedges) which has been identified through a characteristic morphotype known as Cone shaped in previous literature [40]. As this is a well-established name, we will keep it (*nomen conservandum*) [28]. Whereas the presence of this morphotype is scarce to absent appearing only in the AF (<1%) (Table 5).

Dicot phytoliths were scarce to absent (<1%) (Table 5) in the three stratigraphic units. The morphotypes identified are three, Blocky (<1%), Elongate thick (1%) and Spheroid (<1%) (Table 5). Blocky is a common morphotype in leaves of Cyperaceae and Poaceae, but they are also produced by the wood/bark of several dicots and conifers and, when observed in monocots, they usually are interpreted as Bulliform [5,28,41]. Spheroids can also be documented in a wide range of plants, but due to their abundance in some woody plants they are used as an indicator of woody plants [28].

Finally, there is a relatively high amount of indeterminable phytoliths, particularly in the AF and the WS (34 and 25% respectively) (Table 5).

**Other siliceous microremains: Diatoms and sponge spicules**

Diatoms and sponge spicules (Fig 6d) are present in all the stratigraphic units, with the only exception of FL, where no sponge spicules were documented (Table 3). These microremains are related to water environments, and their presence can provide important environmental information [42]. Sponge spicules did not show ornamentation, and they only exhibit their characteristic axial canal. Diatoms are more abundant in WS (3%) and AF (1%) (Table 5). We were able to identify at least two types of diatoms, monoraphideae and biraphideae (probably genera Nitzschioideae). Unfortunately, it was not possible to arrive at species level since their attributions were not fully visible due to orientation and/or visibility.

### Bulk samples

In this section, we will detail the quantification of the bulk sample analyzed and the morphological inventory observed under the microscope.

#### Preservation.

The preservation is overall good with some differences between the stratigraphic units, the percentages of weathered morphotypes are relatively high, 25%, and fragments also raise up to 23% in sample from AF (Table 6). This tendency is completely different in the sample from the WS, where the percentage of weathered morphotypes is 9%, and no fragments were documented (Table 6). Finally, in FL, the percentage of weathered phytoliths is the lowest, < 1%, and fragments are present in 2% (Table 6). Melted phytoliths were only observed in samples from WS, but in low percentages (<1%), while vitrified silica was documented in WS and FL, also in low percentages (1%) (Table 6).

**Table 6:** List of results of phytoliths extractions from BS, including the estimation of phytoliths per gram of sediment, the amount of phytolith morphologically identified, and the percentage of fragments, weathered morphotypes, melted phytoliths, vitrified silica, diatoms, and spicules documented in the three samples.

Sample	#Phytolith per g. of sediment	#Phytolith identified	Fragments	Weathered morphotypes	Melted phytoliths	Vitrified silica	Diatoms	Spicules
Agricultural field	1,600,000	202	23	25	–	–	<1	–
Walking surface	1,500,000	217	20	9	<1	1	4	<1
Floors	2,500,000	219	2	<1	–	1	–	–

#### Phytolith morphological analysis.

The vast majority of the phytoliths morphologically identified in the three samples belong to monocotyledons, specifically to grasses (Poaceae) of the Pooideae subfamily. These plants are represented by Rondel (<53%), Crenate (<5%) and Trapezoids (<2%) (Table 7). Along with Rondel, Bilobate were also observed although their percentage is much lower than the former (<3%), appearing more abundantly in AF (Table 7). Grasses are also represented by Elongate dendritic (5%), E. sinuate (3%), Acute bulbosus (2%) and Bulliform (<1%) (Table 7). In addition to grasses, Cyperaceae plants are only present in AF, although their presence is scarce (<1%) (Table 7).

**Table 7:** Percentage of phytolith morphotypes identified in the BS of the three stratigraphic units, along with their botanical attribution.

Morphotype	Plant attribution	Agricultural fields	Walking surfaces	Floors
Elongate entire	Leaves/stems of monocots/dicots	26	11	6
Elongate ornate	Grasses	<1	–	1
Rondel	Pooideae	53	30	20
Trapezoid		2	2	3
Bilobate	Pooideae/Panicoideae	3	1	<1
Acute bulbosus	Grass leaves	2	<1	1
Bulliform		<1	–	–
Crenate		4	5	–
Elongate sinuate		3	4	<1
Elongate baculate	Grass inflorescence	–	1	–
Elongate dendritic		5	21	63
E. dendritic/dentate		–	18	2
Papillate		–	2	1
Papillate base		–	2	1
Cone shape	Cyperaceae	<1	–	–
Blocky	Dicots wood/bark	–	<1	–
Spheroid		–	<1	–
Elongate thick	Dicots leaves	<1	<1	–
Spheroid echinate	Arecaceae	–	<1	–

**Fig 7:**
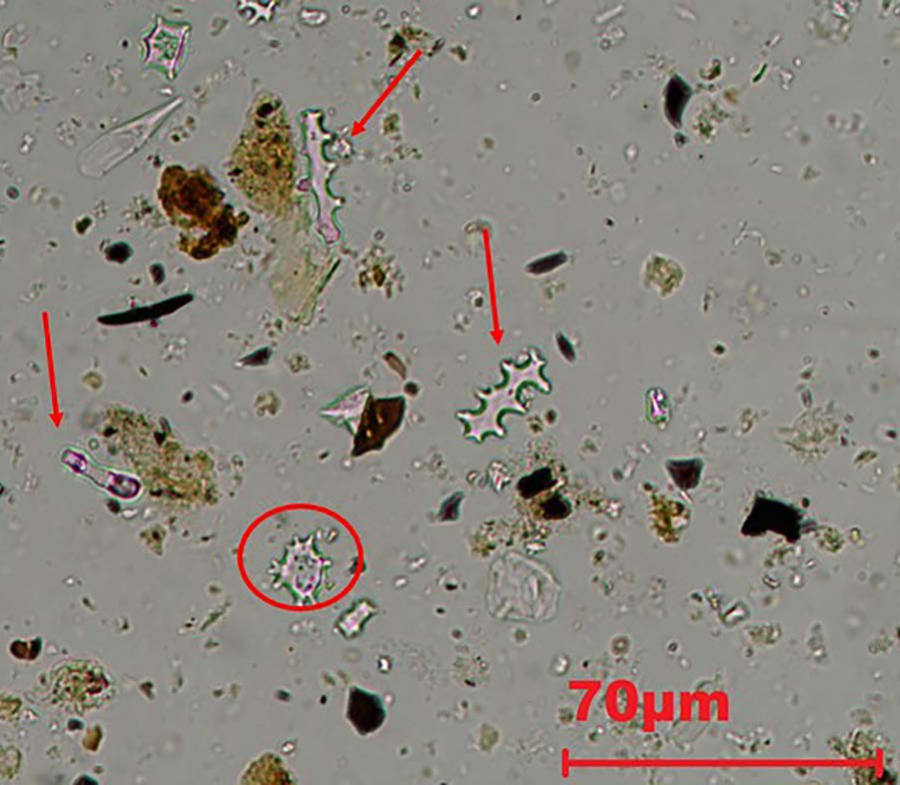
Microphotograph from phytolith extractions of FL sample taken at 500 magnifications. The arrows point to the Elongate dendritic/dentate while the circle shows a Papillate base.

In the case of the sample from FL, the majority of the phytoliths come from grass inflorescences, especially Elongate dendritics which make 63% of the phytolith record. Elongate dendritics are also present in high amounts in the WS, 21% while they represent 5% in the AF (Table 7). E. dendritic/dentate (Fig 7) reach a percentage of 18.4% in the WS and 2% in FL (Table 7). Other morphotypes produced by the inflorescences of grasses such as Papillate and Papillate base (Fig 7) were observed only in FL and WS in amounts of < 2.3% (Table 7).

Finally, phytoliths produced by dicots plants are scarce in the phytolith record and they were documented only in WS and AF, with the presence of Blocky (<1%), Elongate thick (<1%) and Spheroid (<1%) (Table 7). Indetermined phytoliths were not observed in the samples.

**Other siliceous microremains: Diatoms and sponge spicules**

Diatoms and sponge spicules were observed in WS and AF. Diatoms are more abundant in the WS (4%) (Table 6) while in AF these microremains were not observed. It was possible to identify biraphideae (probably genera Nitzschioideae) diatoms, however the attributes were not well preserved, and it was not possible to reach the species level. Conversely, sponge spicules were documented in low amounts (<1%) only in the WS (Table 6).

#### Quantification.

Table 6 lists the estimated amounts of phytoliths per gram of sediment of each sample. Concentrations are similar in AF and WS, bracketed between 1,600,000 and 1,500,000 phytoliths per gram of sediment. Conversely, FL shows higher concentrations of phytoliths per gram of sediment, 2,500,000 (Table 6).

## Discussion

In the following, we will confront the results of both methods obtained by stratigraphic unit and discuss their archaeological significance. After that, we will evaluate the advantages and limitations of combining phytoliths in thin sections and phytoliths in bulk samples.

### Agricultural fields

#### (Post)depositional history.

Despite the overall good preservation of the phytolith assemblage in the STS where weathered morphotypes rises up to 6% (Table 3, S2 Table and Fig 3), in the BS this percentage reaches 25% (Table 6). This difference in the concentration of weathered morphotypes can be explained by the fact that weathered phytoliths can be more difficult to recognize in the STS than in BS. Phytoliths can preserve in a wide range of environments, however they can suffer chemical alteration, even disappear, in sediments with a rather strong alkaline pH (>8.2) [43,44], which is not the case for the DIVA-site where the pH is rather acidic to slightly alkaline. Hence, the high percentage of altered phytoliths suggests that not all phytoliths have the same origin [16]. The presence of burnt phytoliths (autofluorescence and melted phytoliths), along with ashes [18] indicates that burnt plant material was used as a fertilizer spreading them onto the soils, a common practice widely observed in other medieval sites [13,45]. Additionally, burnt phytoliths are less stable than unburnt ones, and thus more prone to dissolution [44], which explains the high percentage of weathered morphotypes observed in the AF.

The fact that only isolated and clustered phytoliths were observed in this stratigraphic unit (Fig 2, S1 Table) further indicates that the plant material deposited in the soil has been released from the organic matter and transported from its original place of deposition [10]. This, together with the presence of phytolith fragments (Table 3, Table 6), suggest mechanical processes (reworking) supporting the idea of an intense agricultural practice carried out at DIVA [18].

#### Plants used.

**Fig 8:**
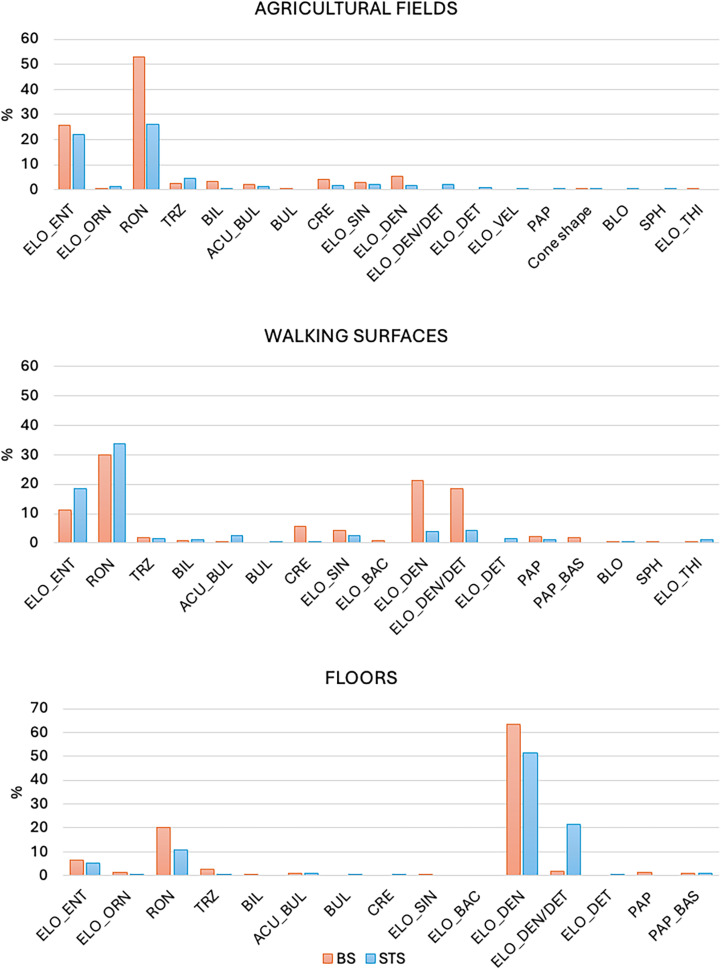
Plots showing the principal morphotypes identified in the STS and the BS.

Phytoliths observed in the AF of DIVA reveal the presence of plants from the C_3_ Pooideae subfamily. In general, STS and BS yielded the same phytolith assemblage characterized by high concentrations of Rondels and Elongate entire, while other morphotypes are less common in the phytolith record. Another observation that comes out from these results is the fact that the percentage of each morphotype is higher in the BS record (Fig 8). A good example in this sense is the differences in the presence of Rondel, which is double in BS (Table 5, Table 7). This can be explained by the presence of indetermined phytoliths in the STS, which rises to 33.7% (Table 7).

As no articulated systems of Elongate dendritics were documented in the AF, no morphometric analyses were realized for this stratigraphic unit. Together with the absence of articulated systems, there is a general scarcity of Elongate dendritics (Table 5, Table 7). The low concentration of this morphotype can be related to the fact that this morphotype is more delicate and hence more prone to dissolution [44]. Another possible explanation is related to the harvest process of the plants, which would prevent the inflorescence of the plant from falling to the ground, in great amounts. Other AF where phytolith analyses were applied also show this tendency [12,46,47].

The presence of Bilobates, although infrequent, can be related to the presence of open grasslands near the site, while the occurrence of Cyperaceae phytoliths suggests the existence of wetlands in the area or close to it. This is consistent with previous data from Antwerp that documented wetlands and open areas nearby [17].

### Walking surfaces

#### (Post)depositional history.

The preservation in this stratigraphic unit is good overall, with low percentages of weathered morphotypes, which is consistent with the acidic to neutral pH of the sediments. Nevertheless, a high concentration of fragmented phytoliths is observed in this stratigraphic unit (Table 3 and Table 6). Similarly, a high concentration of fragmented anthropogenic remains (mainly construction and household waste) was observed in the STS, resulting from trampling [18]. At the same time, the presence of only clusters and isolated phytoliths suggests that after plant material decays it suffers post depositional processes (mainly biogenic, i.e., trampling) that prevent the articulated systems to preserve [2]. Taking all this into consideration it can be suggested that at least part of the plant material in the WS comes from waste material that, after deposition, was subjected to constant trampling resulting in the fragmentation of phytoliths.

#### Plants used*.*

The vast majority of the phytoliths observed in the waking surfaces come from grasses from the Pooideae subfamily. Due to the fact that neither articulated systems nor silica skeletons were observed in STS nor in BS, it was not possible to perform morphometric analysis. Yet, it can be observed that there is a higher amount of inflorescence phytoliths compared to the AF (Table 5 and Table 7). Moreover, the percentage of Elongate dendritics (>21%) in the phytolith assemblage of both STS and BS points to the presence of crops [39]. It is important to note that it is not the grain of the plant that produces these phytoliths, but the bracts in which they are embedded. Normally, the grain is separated from the inflorescence through different processes (winnowing, pounding etc.) that leave a phytolith assemblage that includes husk phytoliths and GSSCP [48]. Therefore, it can be suggested that part of the plant material could come from secondary by-products obtained from the crop processing, that for some reason were rejected and incorporated into the WS as waste, which also includes ashes.

### Floors

#### (Post)depositional history.

**Fig 9:**
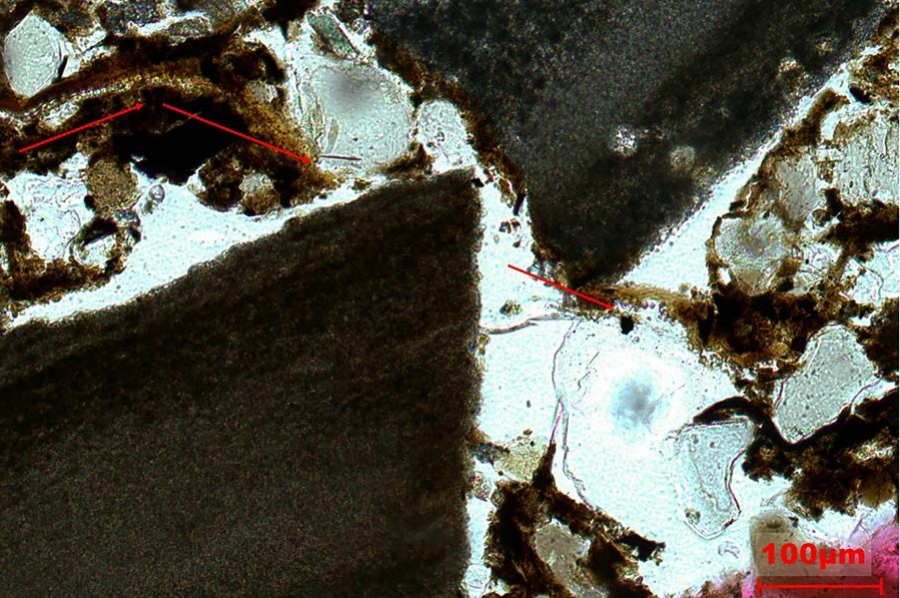
Microphotograph taken at 200 magnifications of an articulated system from sample 196.1. Arrows indicates the location of the articulated system. Note that there is a continuation of the plant tissue on the left side of the picture, revealing this was broken by trampling.

In contrast with the other two stratigraphic units, the majority of phytoliths described in the FL were articulated, e.g., observed in anatomical position (S1 Table, Fig 2). As the phytoliths are still (partly) incorporated within the organic tissues, decay and potential release of the phytoliths in the environment is limited [2,3]. In addition, the articulated phytoliths did not show thermal alteration. This suggests that the plant material did not suffer processing, and it was deposited *in situ*. At the same time, part of the articulated systems show clear traces of trampling (Fig 9), which can be produced by both, humans, and/or animals. As no coprolites were observed [18], it is unlikely that the articulated systems are related to herding activities or omnivore excrements [17]. Even if the coprolites did not preserve and taken into account that dung contains high concentrations of phytoliths, higher quantities of phytoliths are to be expected [31,39,49,50]. With this scenario, it can be argued that the stratigraphic unit related to FL is derived from domestic/artisanal activities, possibly vegetal matting [51], rather than from livestock practices. in this respect, we can also point to the evidence of trampling (Fig 9).

#### Plants used.

The phytolith record in FL significantly differs from the other two stratigraphic units: the Elongate dendritics make up at least 60% of the phytolith assemblage (Table 5 and Fig 8). Thanks to the presence of articulated Elongate dendritics, morphometric analyses were carried out in this stratigraphic unit. Elongate dendritics are useful to attribute to a species level by applying morphometric analyses to measure some of their characteristic shapes (i.e., wave lobes) [25,52–54]. To be able to use morphometric systems it is important to ensure that all the morphotypes analyzed come from the same plant, so all the measurements carried out are related to a single taxon. Hence, morphometric analyses must be carried out over articulated systems or silica skeletons (defined as complete sections of siliciﬁed epidermal tissue in the form of contiguous cells [25]) made of Elongate dendritics [3]. Given the lack of observation of articulated systems or silica skeleton in the bulk samples, these analyses could only be carried out based on the observations done in the STS. Although the statistical analysis does not show conclusive results, the point to the presence of *Avena sp*., which is coherent with the observations under light microscopy of archaeological and reference material, that also point in this direction. Thus, it can be suggested that oat (*Avena sp.)* was used within the domestic context of DIVA.

Oat thrives well in moist and temperate environments, and in northern-west Europe it succeeds better than wheat and is cultivated as principal crop [34]. Even more, it has been documented in many other medieval sites in Belgium [45,55–57].

### Quantifications

Although the estimation of the concentration of phytoliths are not shown in the same scale (phytoliths per mm^3^ in the STS vs phytoliths per gram of sediment in the BS), we expected them to show the same tendency, which is not the case since AF show higher concentrations in STS than in BS, and FL show more concentrations in BS than in STS (Table 3, Table 6). This can be explained by the high variability in the amount of phytoliths observed in the fields of STS, which points to an uneven distribution of phytoliths, probably derived from the different nature of the activities carried out at the site.

### Advantages and limitations of combining phytolith analyses in BS and STS

Although this is not the first time that this type of study has been accomplished [55], this work represents a pioneering study that successfully applies a combined analysis of phytoliths in BS and STS, showing not only the usefulness of this method but also the necessity of combining BS and STS. The possibility of observing the phytolith record within its (micro-)context is key to understanding the (post)depositional processes involved in the deposition of phytoliths. In this sense, this study has demonstrated that it is possible to associate a characteristic phytolith assemblage with a specific activity. It would have been difficult to reach such accuracy by only analyzing the phytoliths from BS, as it was pointed out in previous studies [11]. Moreover, the phytolith assemblages resulting from the observations on STS and BS are similar, confirming that the data set collected through both methods is solid. Moreover, each stratigraphic unit is characterized by a different phytolith spectra, consequence of the different anthropogenic activities carried out on the site. The main difference concerns the amount of indetermined phytoliths, which is high in the STS and absent in the BS, subsequently the information from the BS will fill in the gaps derived from the media of the STS (impossibility to rotate the microremains and their random cutting) that sometimes makes the identification difficult [10,11]. In turn, the observations in the STS allowed to detail the origin of the phytoliths and the processes involved.

At this stage, the main limitation found in the combination of STS and BS is the incoherence in the quantification system and further analysis needs to be performed in this sense to clarify and improve the quantifications.

In summary, the information provided by BS and STS complements each other and permits to obtain a solid data set related to the (micro)context, that allows to go further in the interpretations, enhancing the methodological approach of phytolith studies.

## Conclusions

This study, combining phytoliths in BS and STS provides information that overcomes the limitations each technique has. The application of this method to the Dark Earth of DIVA has drawn a more complete picture on the activities carried out at the site in relation to the use of plants during the period bracketed between the end of the Gallo-Roman empire and the 11^th^ century. Agricultural practices were focused on the cultivation of plants from the Pooideae subfamily and were developed with a previous preparation of the soil by spreading ashes to fertilize it. The intense agricultural practices are revealed by the fragmentation of the phytoliths and the absence of articulated systems. In the walking surfaces, secondary by-products of crops are part of the waste, which is object of continuous trampling, while on the earthen floor surfaces secondary by-products of oat are part of the flooring material that got subsequently trampled. Although further analysis needs to be done in order to solve the limitations on the quantitative analyses performed on the STS, our results demonstrate the necessity of combining phytolith analysis in STS and BS.

## Supporting information

S1 Table Percentages of each distribution pattern for each stratigraphic unit. (XLSX)

S2 Table Percentages VPC index for each stratigraphic unit. (XLSX)
